# Role of Allogeneic Hematopoietic Stem Cell Transplantation in Adult Patients with Acute Lymphoblastic Leukemia

**DOI:** 10.4084/MJHID.2014.065

**Published:** 2014-11-01

**Authors:** Federico Lussana, Alessandro Rambaldi

**Affiliations:** 1 Hematology and Bone Marrow Transplant Unit, Azienda Ospedaliera Papa Giovanni XXIII, Bergamo, Italy.

## Abstract

Adult acute lymphoblastic leukemia (ALL) is a heterogeneous disease, due to the expression of different biological and clinical risk factors, for which allogeneic stem cell transplantation (alloHSCT) is an effective consolidation therapy. The non-relapse mortality of alloHSCT remains significantly higher compared with that of conventional chemotherapy. Therefore, one of the main challenges in the care of ALL is to establish a more precise prognostic definition to select patients who could take advantage from an alloHSCT. Currently, the use of minimal residual disease following induction and early consolidation therapy has improved the prognostic accuracy in defining ALL risk class. In Philadelphia-positive ALL, the introduction of tyrosine kinase inhibitors pre and post alloHSCT appears to improve outcomes significantly and, in the absence of specially designed clinical trials, alloHSCT remains the most effective post-remission therapy. Nowadays, alloHSCT can be performed according to various modalities encompassing the use of different conditioning regimens, as well as distinct donors and stem cell source, with a significant accessibility to transplant.

## Introduction

Modern intensive induction chemotherapy allows most adult patients with acute lymphoblastic leukemia (ALL) to achieve a complete hematologic remission (CR). However, only 40% of patients survive five years or more.^1^–^4^ Allogeneic hematopoietic stem cell transplantation (alloHSCT) is an effective post-remission treatment in patients with ALL; however, its remarkable curative potential is often counterbalanced by a high incidence of post-transplant complications that lead to a high non-relapse mortality (NRM). Chronic graft-versus-host disease (GVHD) with a related poor quality of life represents an additional severe concern, so that the optimal timing and use of this treatment modality remains an issue of debate. Thus, it is crucial to identify patients who have high chances of cure with standard therapy and those for whom alloHSCT is the only possible post-remission therapy. In this regard, a risk-adapted strategy, using clinical and/or biological features, such as age, white cell count, time to obtain CR, disease immunophenotype, cytogenetics, and molecular abnormalities, may help in selecting patients at highest risk for relapse, who may benefit from alloHSCT. Moreover, evidence is growing that the evaluation of minimal residual disease (MRD) can further improve the prognostic accuracy in defining ALL risk classes. In this review, we update the indications for an alloHSCT in adult ALL patients. We also discuss the issue of the conditioning regimens before transplant as well as the most appropriate stem cell source.

## Which Patients Should Have a Transplant?

### Clinical and biological risk stratification

At diagnosis, prognostic factors can be defined as patient or disease related. Among these latter white blood cell count (WBC) and some, well defined, immunologic, cytogenetic or molecular profiles reflect the extensive biologic heterogeneity of ALL.^5^ A high WBC count at diagnosis (greater than 30×10^9^/L for B lineage ALL and 100×10^9^/L for T-lineage ALL) is associated with a poor prognosis.^6^–^9^ Similarly, a very immature phenotypes, such as pro-B or pro and pre T, and mature T phenotypes (EGIL BI and TI/TII/TIV) are considered prognosticator adverse.^10^,^11^ Some cytogenetic abnormalities such as t(9;22)(q34;q11), t(4;11)(q21;q23), t(8;14)(q24.1;q32), the presence of a complex karyotype (defined as ≥ 5 chromosomal abnormalities) or low hypodiploidy/near triploidy, are similarly associated with an adverse prognosis.^12^,^13^ Age remains the most important clinical risk factor and overall survival (OS) dramatically decreases with an increasing age. Young adults (younger than 35 years) may have a very good OS (up to 60% or more) if they are appropriately treated according to intensive pediatric protocols. On the contrary older adults (older than 55) have a probability of survival of 20% at 3 years with a disease-free survival (DFS) rate lower than 20% if no transplant options are offered as post-remission therapy.^6^,^7^,^14^–^17^ The above reported prognostic factors are summarized in Table 1.

### Risk class and alloHSCT

Several studies have shown a potential benefit for alloHSCT over conventional chemotherapy for patients with high-risk (HR) disease. The French multi-center randomized trial LALA-87 found that only patients with HR features have better OS and DFS with alloHSCT, while patients with standard-risk (SR) had no significant advantage of alloHSCT over chemotherapy or autologous HSCT.^18^ This was confirmed by the same group in a larger study (LALA-94), that stratified only HR patients with donors to alloHSC, showing a better result in patients transplanted (5-year DFS 45% in transplanted compared to 23% in patients without donor, p=0.007).^1^ Similarly, the GOELAL02 trial showed an improved six year OS in the HR patients undergoing alloHSCT compared to patients without donor (6-year OS 75% vs. 40%, p=0.0027, respectively).^16^ Other studies suggested that alloHSCT in HR patients in first CR (CR1) offers good survival rates ranging from 40 to 60%.^19^–^23^ In contrast to the former results, there have been a number of studies, including the EORTC ALL3 trial^24^ or the Spanish PETHEMA ALL-93 trial,^17^ that failed to prove that alloHSCT produces a better outcome than autologous HSCT or chemotherapy in adults with HR ALL. More recently, the large MRC/ECOG trial^7^, in which the outcomes were estimated from a donor versus no donor analysis of 2000 patients between 15 and 55 years old, showed that an allogeneic transplant resulted in better disease control compared to chemotherapy or autologous transplant. However, surprisingly, in this study the 5-year OS was significantly improved in SR patients (62% vs52%, p=0.02), while in the HR group the reduction of relapse rate was offset by a high non relapse mortality (NRM) (at 2 years 36% vs. 22 with conventional therapy) with a 5-year OS of 41% vs 35%, p=0.2.

### Meta-analyses and unsolved issues

The uncertain results in evaluating the real benefit of the allogeneic transplant in HR patients has been highlighted by several meta-analyses performed over the past few years.^25^–^27^ In the most recent one, including data from 13 studies involving 2962 patients (excluding Ph+ patients), the age at transplant turned out to be again the most important prognostic factor, since an actual survival benefit was reported only for patients < 35 years of age (OR 0.79; 95% CI, 0.70–0.90) because of the higher absolute risk of NRM for older patients.^28^ There are several explanations for these controversial results, the most likely being that some of the analyzed trials were either numerically underpowered or gave variable definitions of “high-risk” ALL.

All in all, these controversial results highlight the fact that in adult ALL, classic prognostic factors have a limited accuracy,^2^,^3^,^6^,^12^,^29^ since a significant proportion of SR patients (up to 40% to 50%), treated with standard chemotherapy, will eventually relapse and conversely, 20% to 25% of HR patients will not relapse even in the absence of an alloHSCT.^1^,^7^,^16^,^17^,^30^

### Minimal residual disease

In addition to the above mentioned clinical and biologic prognostic features identified at diagnosis, the most important prognostic factor remains the sensitivity to chemotherapy. This crucial factor can be evaluated by several ways which include time to achieve complete remission, time to leukemic blast cell clearance and, particularly in childhood ALL, the initial response to prednisone. However, most recently, a number of studies performed in children and adults have clearly shown how quantitative measurements of MRD at various time points during treatment provide the most accurate estimate of chemosensitivity. The definition of MRD either by immunophenotype or molecular biology, implies the ability to identify the persistence of a very low number of leukemic cells in patients otherwise considered in hematologic remission. The persistence of such MRD is an indicator of intrinsic drug resistance that can herald overt hematologic relapse.^9^,^31^–^35^ The persistence after ten weeks of treatment of MRD at a level exceeding 10^−4^ or 10^−3^ identifies a condition of high or very high risk, respectively, for relapse. Recently, a panel of experts summarized how to best assess MRD analysis that can be made using either real-time quantitative polymerase chain reaction (RQ-PCR) or multichannel flow cytometry,^36^ which is normally applicable in the vast majority of patients.^5^ The use of MRD has been shown to be highly promising prognostic and decisional tool during the treatment program of ALL patients. The German Multicenter Study Group for Adult ALL (GMALL), in 196 SR patients, identified different MRD groups with 3-year relapse rates ranging from 0% to 94%.^33^ Data from the MRC/ECOG trial^35^ and Northern Italy Leukemia Group (NILG)^9^ confirmed that MRD was the most significant risk factor for relapse, with a favorable prognosis in patients responsive to chemotherapy obtaining a MRD negativity and a poor leukemia-free survival in patients MRD positive. The GMALL group recently published the largest cohort of MRD data in adults, corroborating the evidence that the measurement of MRD allows the identification of subgroup of patients with an inadequate initial response and a high rate of relapse.^37^ Finally, new data from Spanish PETHEMA group showed in multivariable analysis that reduced MRD clearance was the only prognostic factor for an unfavorable DFS and OS.^38^

Table 2 summarizes the association between MRD status and clinical outcomes in ALL patients, reporting data from the larger prospective studies.

Overall, these data provide evidence of utility of introducing MRD analysis for the identification of patients at high risk of relapse who may benefit from early transplantation, despite a morphologic remission and the absence of clinico-biological risk factors and, conversely, for the identification of a group of patients who are sensitive to chemotherapy, achieving non-detectable level of MRD, who probably do not need transplantation as consolidation therapy also when risk factors are present. The most relevant and urgent challenges into future investigations about MRD are: a) to determine the most predictive time point for the measurement and the threshold during and after treatment b) to identify the best strategy in patients with high MRD before allogeneic transplantation, such as the need of further therapy to reduce tumor load before transplant c) to demonstrate the utility and efficacy of tapering or withdrawal of immunosuppressive therapy or use of donor lymphocyte infusion (DLI) in patients in molecular relapse after transplant; d) to demonstrate the efficacy of an MRD-guided choice in avoiding the alloHSCT in HR patients with no detectable MRD.

### Allogeneic transplant for patients with Philadelphia-positive (Ph+) ALL

Historically, Ph+ ALL marked the most unfavorable subgroup of adult ALL and in the pre-tyrosine kinase inhibitors (TKIs) era, the overall survival observed in unselected series of patients was less than 25% even when alloHSCT was offered whenever possible.^5^,^39^ The incorporation of TKIs in the standard ALL therapy significantly changed the outcomes of ALL, increasing the remission induction rates as well as the depth of remission,^40^–^43^ allowing an improved cure rate. Unfortunately, although almost all patients receiving single agent imatinib or dasatinib achieve a hematologic CR,^44^,^45^ it is likely that at least during the consolidation phase, TKIs should be used in combination with chemotherapy.^46^,^47^ The use of refined programs with first/second generation TKIs and chemotherapy together with alloHSCT allow up to 50% of all patients to be cured.^40^,^48^,^49^ A positive impact of imatinib on the clinical outcome of alloHSCT has been reported early after its therapeutic use in Ph+ ALL^50^ and this result has been recently confirmed by the Japanese Adult Leukemia Study Group that compared the alloHSCT outcomes between 542 patients who received imatinib before alloHSCT during the initial complete remission period (imatinib cohort) and 196 patients who did not receive imatinib (non-imatinib cohort). The 5-year OS was significantly higher in the imatinib cohort than in the non-imatinib cohort (59% vs 38%; p <0.001).^51^ There is little information on the efficacy of TKIs administration after alloHSCT. Recently, Pfeifer et al. compared the tolerability and efficacy of post-transplant imatinib, administered either prophylactically or following detection of MRD, showing that prophylactic imatinib significantly reduced the incidence of molecular recurrence after alloHSCT compared with MRD-triggered imatinib (40% vs 69%; P=0.046).^52^ Ram et al reported that imatinib, given after reduced intensity conditioning (RIC) alloHSCT conditioned with fludarabine and low dose total body irradiation (TBI), was associated with significantly reduced mortality in univariate analysis, although the effect on relapse was not statistically significant.^53^ Finally, data from European Society for Blood and Marrow Transplantation (EBMT) registry^54^ showed that the introduction of TKIs pre and post alloHSCT significantly improved outcomes of Ph+ ALL patients. Of note, in this analysis the Authors described a significant association between the use of TKIs post-transplant and a low cumulative incidence of chronic GVHD, including its extensive form. In general, the available data suggest that TKIs administration at time of induction should be considered as a mainstay treatment of Ph+ ALL patients, because it increases initial remission rates with a beneficial impact in feasibility of alloHSCT and also improves outcomes after alloHSCT, while the impact of TKIs-based therapy on long term outcome after alloHSCT remains unclear.

### Impact of minimal residual disease on allogeneic transplantation

It must be kept in mind that patients undergoing allogeneic transplantation with measurable level of minimal residual disease clearly show an inferior outcome after transplant due to a significant increased risk of leukemia relapse.^55^–^58^ This implies that, whenever possible, patients selected for allogeneic transplant should be considered for newer experimental treatment strategies able to achieve such a molecular remission before the transplant is performed. Among such innovative treatments for the treatment of minimal residual disease, blinatumomab, the first member of a novel class of T cell-engaging, bispecific single-chain (BiTE) antibodies (it engages T cells for redirected lysis of CD19+ target cells) showed very encouraging results.^59^ The crucial prognostic role of MRD has been demonstrated also in the case of Ph+ patients for whom the effort to achieve a convincing molecular CR should be pursued. In this respect, Ponatinib (a third generation TKI) has been recently shown to overcome the pharmacologic resistance mediated by some mutations of the BCR/ABL protein such as the T315I, and achieve impressive frequency of molecular CR.^60^

### Allogeneic transplant for relapse or refractory ALL patients

ALL is primary refractory to chemotherapy in less than 10% of the cases. According to a few large studies from MRC/ECOG 61 and LALA groups^62^ the salvage treatment after relapse may induce an hematologic remission in some patients but the long term outcome is usually dismal, although more intensive re-induction chemotherapy programs followed by an alloHSCT may achieve durable remissions.^63^ Doney et al. reported a long-term DFS of 24% for patients transplanted after achieving a second remission, and of only 9% in those transplanted with refractory disease, confirming that in most cases salvage after relapse is not feasible even with transplantation.^64^ In this setting the use of blinatumomab^65^ and other innovative drugs such as inotuzumab^66^ or ponatinib^47^ may open the door to effective rescue treatments which may significantly increase the effective role of allogeneic transplant for this unlucky group of patients.

## How to Perform an Allogeneic Transplant

It is now clear that alloHSCT represents a very effective post-remission therapy with high chances of achieving a long-term disease control in patients at highest risk for relapse. Nowadays, this procedure can be performed according to remarkably different modalities encompassing the use of different conditioning regimens, as well as different donors and stem cell source.

## Which is the Optimal Transplant Regimen?

### Myeloablative conditioning (MAC)

Myeloablative conditioning regimens based on a combination of cyclophosphamide (Cy) (120 mg/kg) and total body irradiation (TBI), ranging from 12 up to 13.2 Gy, are still currently used as standard preparative regimens for ALL, due to the intrinsic and powerful anti-leukemic activity and the peculiar ability to eradicate leukemic cells within the central nervous system and testicles^67^,^68^ of radiotherapy. However, in order to reduce radiation related toxicities other regimens have been developed. Substituting busulfan (BU) for TBI showed comparable OS, relapse rate and DFS to the TBI regimen, but until conversion from oral to IV BU the number of serious side effects, such as hepatic veno-occlusive disease (VOD) or hemorrhagic cystitis was high.^69^,^70^ Recently, the combination regimen of IV BU/Cy in CR1 patients showed a 3 year OS of 66% with a relapse rate of 40% and a decreased transplant related mortality (TRM).^71^ The group at the City of Hope National Medical Center evaluated the substitution of Cy with etoposide (VP-16) in combination with fractioned TBI (13.2 Gy) reporting a significant activity also in patients with advanced ALL with a DFS of 57% and a relapse rate of 32%.^72^ These interesting results were confirmed in a subsequent trial conducted by Southwest Oncology Group.^73^ Finally, Marks et al. recently published a comparative study of TBI combined with either Cy or VP-16.^74^ In this study 4 groups were compared based on the radiation dose: Cy-TBI<13Gy, Cy-TBI>13Gy, VP-16-TBI <13Gy and VP-16-TBI >13Gy. No difference in OS, DFS and TRM was observed by conditioning regimen in CR1 patients, while an advantage in substituting VP-16 for Cy or, when Cy is used, in increasing the TBI dose > 13 Gy was reported for patients in CR2.^74^

In conclusion, the standard MAC are regimens based on TBI combined with Cy or VP-16, although to avoid TBI toxicities, the use of a regimen containing IV BU and Cy is considered a valid a safe alternative. Based on EBMT registry data,^71^ the use of regimens using IV BU is increasing.

### Reduced intensity conditioning (RIC)

The procedure of RIC transplant has been established in acute myeloid leukemia setting, while the experience in ALL is limited with no randomized trial comparing RIC to MAC. Two large registry studies have demonstrated the feasibility and potential efficacy of RIC for ALL. The retrospective study from the EBMT^75^ compared the outcome of 576 adult ALL patients who received a RIC (n=127) or MAC (n=449) alloHSCT in CR. As expected, the multivariate analysis showed that RIC patients experienced a decreased risk for NRM compared to MAC (hazard ratio (HR) 1.98, p=0.0001), but a higher relapse rate (HR 0.59, p=0.03). However, in multivariate analysis, the type of conditioning regimen (RIC vs. MAC) was not significantly associated with leukemia free survival (LFS) (HR 0.84, p=0.23). Data from a second large registry of International Bone Marrow Transplant Registry (IBMTR) confirmed the feasibility of RIC with OS at 3 years of 45%.^76^ In keeping with registry data, other small studies, reporting single Institution experience, demonstrated RIC feasible with good outcomes with OS over 50% and TRM between 10 and 20%.^53^,^77^–^79^ Of note, in patients transplanted in CR2, or not in remission RIC regimen seems to have a limited benefit.^53^,^80^

In conclusion, even if these results did not allow definitive conclusions, due to the absence of randomized clinical trial comparing RIC vs MAC, they suggest that RIC could be a reasonable therapeutic option for ALL patients not eligible for MAC.

## Is there Evidence about the Optimal Donor Cell Source?

Matched related donors are the ideal choice for alloHSCT due to lower incidence and severity of GVHD, but only about 30% of patients who need a transplant have an identical sibling donor. Advances in high-resolution human leukocyte antigen (HLA) typing and improvements in GVHD prophylaxis have made possible the use of alternative donor sources of stem cells, including matched unrelated, cord blood and haploidentical donor. According to a retrospective analysis of the IBMTR registry in 672 ALL patients a similar incidence of relapse and NRM after alloHSCT from matched unrelated donor (MUD) and matched related was observed with an OS approximately of 50% at 5 years.^81^ Recent retrospective studies comparing unrelated with sibling donors confirmed comparable outcomes: Kiehl et al. reported no significant difference in 5 years DFS in 221 patients in CR1 who underwent MUD vs sibling donor transplant (45% vs 42%, respectively)^82^ and a larger study of 1139 patients^83^ showed no significant difference between related and unrelated alloHSCT (at 4 years OS 65% vs 62% in related vs unrelated, respectively p=0.19).

For patients without MUD donors, the use of unrelated cord blood (UCB) and haploidentical donor are under clinical investigations. According to IBMTR registry data of 150 UCB transplants, of which 45 in patients with ALL, OS was 26% in UCB compared to 35% in MUD, with a higher TRM.^84^ The EBMT and eurocord data showed similar results between UCB group (including 53 patients with ALL of 98) and MUD group (including 267 ALL patients of 584) in terms of OS, NRM and relapse rate.^85^ Several recent small studies confirmed encouraging results with the use of UCB reporting no significant difference in OS and DFS between patients who underwent a UCB vs MUD transplantation.^86^–^88^ Finally, a very recent study comparing outcomes of 116 UCB transplants with 686 unrelated adult donor found that the survival of UCB graft recipients at 3 years (44%) was similar to that of recipients of matched or mismatched unrelated adult donor grafts (44% and 43%, respectively).^89^ The use of haploidentical HSCT should be still considered an experimental procedure. Early results obtained with an ex vivo T cell depletion approach showed dismal results.^90^ More recently, and in a pediatric setting, more attractive results have been reported and it is tempting to speculate that the different laboratory procedures for ex vivo T cell depletion or the use of post-transplantation cyclophosphamide may be responsible for these better results.^91^–^93^

In general, the results of the above discussed studies demonstrate the feasibility of alloHSCT from alternative source of stem cells and, in particular, the use of UCB should be considered a valid alternative source of stem cells for adults with ALL in the absence of an identical sibling donor or MUD. However, further studies specifically designed to test prospectively whether or not alternative cell sources are effective and safe for adult ALL need to be developed.

## Conclusions

**Figure 1:**
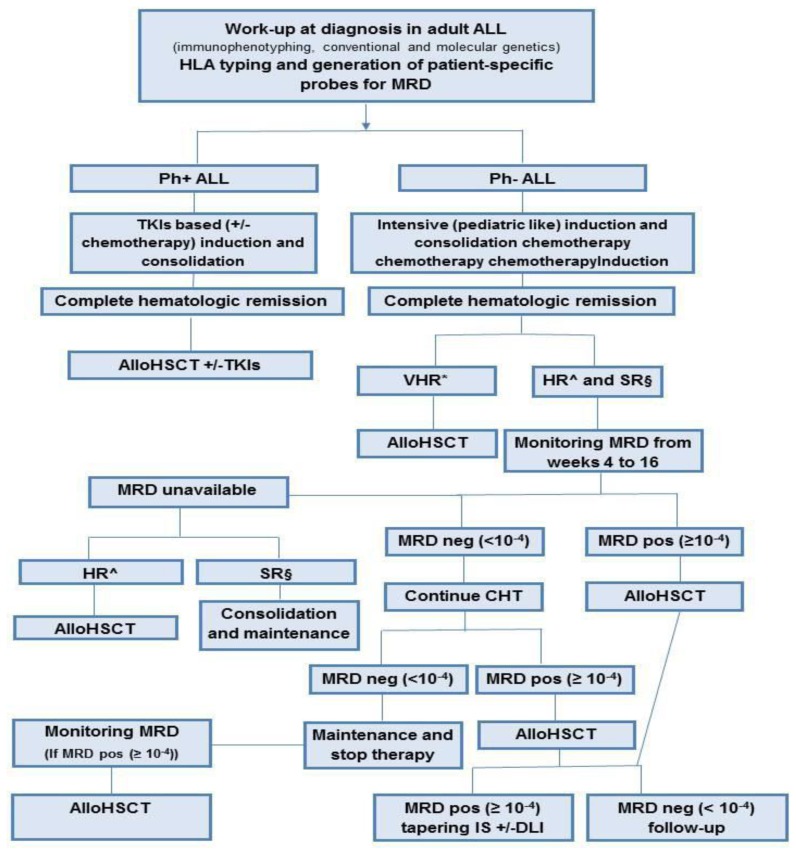
Summary of our suggestions for the treatment of adult acute lymphoblastic leukemia. ALL=acute lymphoblastic leukemia; HLA=human leukocyte antigen; MRD=minimal residual disease; Ph+=Philadelphia-positive; Ph-=Philadelphia-negative; TKIs=tyrosine kinase inhibitors; alloHSCT= allogeneic stem cell transplantation; CHT=chemotherapy; IS= immunosuppressive; DLI= donor lymphocyte infusion. * VHR (very high risk)= cytogenetics (t(4;11), t(8;14), t(1;19), abn 11q23, +8, −7, del6q, low hypodiploidy with 30–39 chromosomes, near triploidy with 60–78 chromosomes, complex with ≥5 unrelated anomalies); molecular genetics (KMT2A rearrangements, BCR-ABL1-like ALL (CLRF2/JAK mutations), IKZF1 deletion (B-ALL); wild-type NOTCH1/FXBW7, altered RAS/PTEN (T-ALL); dysfunctional apoptosis/proliferation mechanisms (p53, Caspases, MYC); MRD>10^−3^ ^ HR (high risk)= age≥ 35 years; white blood cell > 30×109/L for B cell phenotype >100×109/L for T cell phenotype; Very immature phenotypes, such as pro-B or pro and pre T phenotypes and mature-T (European Group for Immunophenotyping of Leukemias BI and TI/TII/TIV); late complete remission; minimal residual disease MRD> 10^−4^ § SR (standard risk): none of the indicated risk factors

Adult ALL is a heterogeneous disease for which alloHSCT is an effective consolidation therapy. The non-relapse mortality of alloHSCT remains significantly high compared with that of conventional chemotherapy, thus, one of the main challenges in the care of ALL is to establish a more precise prognostic definition for selection of patients at higher risk for relapse to make better therapeutic decisions. In this context, a risk-adapted strategy is critical and the use of MRD has shown promising results in establishing more precise prognostic definitions of patients at highest risk for relapse. MRD status must be considered also after alloHSCT for deciding additional treatments to prevent relapse. In Ph+ ALL the introduction of TKI pre and post alloHSCT appears to significantly improve outcomes and, in the absence of specifically designed clinical trials, it remains the most effective post-reemission therapy for this HR ALL. An appropriate timing for alloHSCT is crucial to obtain better results, given that the prognosis of relapsed ALL is very poor and the possibility of achieving CR2 is uncertain. Thus, in most patients HLA typing should be performed at diagnosis for considering transplant options early during the treatment. The development of RIC regimens and the possibility of using alternative sources of stem cells, such as MUD and UCB grafts, facilitate the possibility of transplant in a wider range of patients including those without a family donor and also elderly patients and those with comorbid conditions. Balancing the risks of relapse rate against the potential adverse effects of alloHSCT, in Figure 1 we summarized our suggestions for treatment of adult ALL. As discussed in the review, our strategy was derived also from observational databases and from small retrospective single Institution experience and is therefore subject to debate. Further prospective clinical studies are needed to obtain definitive and reliable data and, considering the low incidence of ALL in adults; these efforts should probably be made within collaborative multicenter studies.

## Figures and Tables

**Table 1:** Clinical and biological risk factor at diagnosis in adult ALL

Risk factors	Very high risk	High risk Any of the following:
Age≥ 35 years		✓
WBC > 30×10^9^/L for B cell phenotype >100×10^9^/L for T cell phenotype		✓
Very immature phenotypes, such as pro-B or pro and pre T phenotypes and mature-T (EGIL BI and TI/TII/TIV)		✓
Cytogenetics: t (9;22) prior to tyrosine kinase inhibitors; t(4;11), t(8;14), t(1;19), abn 11q23, +8, −7, del6q, low hypodiploidy with 30–39 chromosomes, near triploidy with 60–78 chromosomes, complex with ≥5 unrelated anomalies	✓	
Molecular genetics: BCR-ABL1 rearrangement prior to tyrosine kinase inhibitors, KMT2A rearrangements, BCR-ABL1-like ALL (CLRF2/JAK mutations), IKZF1 deletion (B-ALL); wild-type NOTCH1/FXBW7, altered RAS/PTEN (T-ALL); dysfunctional apoptosis/proliferation mechanisms (p53, Caspases, MYC)	✓	
Late CR		✓
MRD> 10^−4^		✓
MRD> 10^−3^	✓	

Standard risk: none of the indicated risk factors. WBC=white blood cell; EGIL= European Group for Immunophenotyping of Leukemias; CR=complete remission; MRD=minimal residual disease.

**Table 2:** Prognostic impact of MRD in prospective studies of adult ALL

Study, year (reference)	Patients	MRD method	MRD study (level for negativity)	Outcomes MRD negative	Outcomes MRD positive
**Bassan, R 2009 (**13**)**	280 adult ALL patients	RQ-PCR	MRD≤10^−4^	5-year OS 75% 5-year DFS 72%	5-year OS 33% 5-year DFS 14%
**Gökbuget, N 2012 (**37**)**	580 Ph- adult ALL patients	RQ-PCR	MRD≤10^−4^	5-year OS 80% 5-year DFS 67%	5-year OS 42% 5-year DFS 25%
**Ribera, JM, 2014 (**38**)**	326 HR Ph- adult ALL patients	Flow cytometry	Patients with MRD< 1 × 10^−3^ after induction and < 5 × 10^−4^ after early consolidation	5-year OS 56% 5-year DFS 51%	5-year OS 17% 5-year DFS 0%

MRD=minimal residual disease; DFS=disease free survival; OS= overall survival; RQ-PCR= Real-time quantitative PCR
